# Role for the PIP_2_‐binding protein myristoylated alanine‐rich C‐kinase substrate in vascular tissue: A novel therapeutic target for cardiovascular disease

**DOI:** 10.1002/ccs3.12052

**Published:** 2024-10-02

**Authors:** Anthony P. Albert, Kazi S. Jahan, Harry Z. E. Greenberg, Yousif A. Shamsaldeen

**Affiliations:** ^1^ Vascular Biology Section Cardiovascular & Genomics Research Institute City St. George's University of London London UK; ^2^ School of Applied Sciences University of Brighton Brighton UK

**Keywords:** contractility, MARCKS, migration, permeability, PIP_2_, proliferation, vascular endothelial cells, vascular smooth muscle cells

## Abstract

In vascular smooth muscle cells (VSMCs) and vascular endothelial cells (VECs), phosphatidylinositol 4,5‐bisphosphate (PIP_2_) acts as a substrate for phospholipase C (PLC)‐ and phosphoinositol 3‐kinase (PI3K)‐mediated signaling pathways and an unmodified ligand at ion channels and other macromolecules, which are key processes in the regulation of cell physiological and pathological phenotypes. It is envisaged that these distinct roles of PIP_2_ are achieved by PIP_2_‐binding proteins, which act as PIP_2_ buffers to produce discrete pools of PIP_2_ that permits targeted release within the cell. This review discusses evidence for the expression, cell distribution, and role of myristoylated alanine‐rich C‐kinase substrate (MARCKS), a PIP_2_‐binding protein, in cellular signaling and function of VSMCs. The review indicates the possibilities for MARCKS as a therapeutic target for vascular disease involving dysfunctional cell proliferation and migration, endothelial barrier permeability, and vascular contractility such as atherosclerosis, systemic and pulmonary hypertension, and sepsis.

## Graphical abstract

Representation of the role of MARCKS in VSMCs and VECs, showing that unstimulated MARCKS is expressed at the plasma membrane where it forms electrostatic interactions with PIP_2_. Cell stimulation by G‐protein‐coupled and tyrosine kinase receptors induces to phosphorylation MARCKS by various mechanisms including protein kinase C (PKC), Ca^2+^‐calmodulin (CaM), and H_2_O_2_ production, leading to the translocation of MARCKS from the plasma membrane into the cytoplasm and release of PIP_2_ into the local environment where it interacts with transmembrane proteins. Both phosphorylation and translocation of MARCKS and PIP_2_–protein interactions were associated with changes in cell function such as proliferation and migration, endothelial barrier permeability, and smooth muscle contractility.

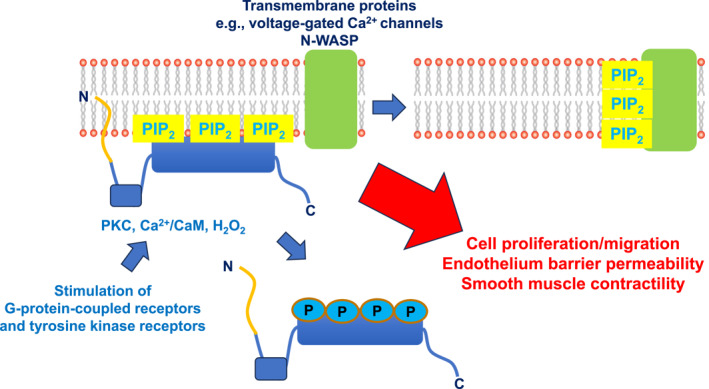

## 1 INTRODUCTION

Phosphatidylinositol 4,5‐bisphosphate (PIP_2_) is a negatively charged phospholipid composed of two fatty acid chains coupled to a water‐soluble inositol head group, which is phosphorylated at its 4′ and 5′ positions. PIP_2_ is mainly found on the inner leaflet of the plasma membrane where it makes up approximately 1% of the total cell phospholipid content and acts as the primary substrate for phospholipase C (PLC)‐mediated generation of inositol 1,4,5‐trisphosphate (IP_3_) and diacylglycerol (DAG) and phosphoinositol 3‐kinase (PI3K)‐mediated generation of phosphoinositol 3,4,5‐trisphosphate (PI(3,4,5)P_3_).^1^, ^2^, ^3^ These PLC‐ and PI3K‐mediated pathways represent ubiquitous signal transduction systems, which are stimulated by an array of external stimuli acting at plasmalemmal receptors, including G‐protein‐coupled and tyrosine kinase receptors that regulate multiple physiological and pathological cellular processes throughout the body.

In vascular smooth muscle cells (VSMCs) and vascular endothelium cells (VECs), stimulation of PLC‐ and PI3K‐mediated pathways are central to blood vessel function including vasoconstrictor‐induced contractility and nitric oxide (NO)‐induced vasodilatation through IP_3_‐mediated Ca^2+^ release mediated by IP_3_ receptors located on the sarcoplasmic reticulum and regulation of DAG‐ and PIP_3_‐mediated downstream molecules such as protein kinase C (PKC) and cation, Cl^−^, and K^+^ channel subtypes that modulate membrane potential.^4^, ^5^, ^6^, ^7^ In VSMCs, vasoconstrictor‐induced membrane depolarization activates voltage‐gated Ca^2+^ channels (VGCCs), with the associated rise in intracellular Ca^2+^ contraction ([Ca^2+^]_i_) leading to the activation of Ca^2+^‐calmodulin (CaM), myosin light chain kinase (MLCK), interactions between myosin and actin, and contraction.^6^ In addition, growth factors such as platelet‐derived growth factor (PDGF) stimulate tyrosine kinase receptors that are coupled to PLC‐ and PI3K‐mediated pathways that are implicated in switching of VSMCs and VECs from physiological into synthetic, pathological phenotypes are associated with changes in cell growth, proliferation and migration, endothelial barrier permeability, and vascular contractility and are linked to vascular diseases involving excessive vasoconstriction and cell proliferation such as systemic and pulmonary hypertension and atherosclerosis and excessive vasodilatation such as sepsis.^4^, ^8^

In addition to its classical role as a substrate for PLC‐ and PI3K‐mediated activity, PIP_2_ also acts as an unmodified ligand to directly regulate cellular proteins such as ion channels and transporters through regulating membrane targeting, enzyme activation, cytoskeletal arrangement, and membrane trafficking.^1^, ^2^, ^3^, ^9^, ^10^ Many of these signaling pathways are critical components in regulating membrane potential, Ca^2+^ influx pathways, and intracellular Ca^2+^ levels in VSMCs and VECs that are important in regulating phenotypic switching.^8^ This raises a paradox; how can PIP_2_ act as both substrate and unmodified ligand to regulate different cell signaling pathways and functions?

This review addresses this question by discussing evidence indicating that myristoylated alanine‐rich C‐kinase substrate (MARCKS), a PIP_2_‐binding protein, has an important role in regulating PIP_2_‐mediated processes in VSMCs, including proliferation, migration, and contractility and in vascular endothelial cells (VECs) through regulation of L‐arginine transport, cell movement and endothelial permeability.

## 2 TARGET‐SPECIFIC PIP_2_ SIGNALING

An explanation for how PIP_2_ might act as a substrate for PLC‐ and PI3K‐mediated pathways and also as an unmodified ligand is the existence of independent pools of PIP_2_ within the cell, which are proposed to be produced through several different mechanisms: (1) Interactions between PIP_2_ and molecules through hydrogen bonding, (2) PIP_2_ accumulation at cholesterol‐rich membrane rafts, (3) localized production of PIP_2_, (4) differential areas of PIP_2_ produced as a consequence of membrane curvature, and (5) PIP_2_‐binding proteins that produce electrostatic sequestration of PIP_2_ through interactions with basic amino acid residues present in their structure.^1^, ^9^, ^11^, ^12^, ^13^ The latter electrostatic sequestration model is an attractive hypothesis as this would allow PIP_2_ to be retained in a local environment, preventing PIP_2_ from rapidly diffusing away from its site of action and potentially being metabolized.

PIP_2_‐binding proteins are divided into two groups.^1^ First, proteins with a known structure that bind PIP_2_ with high specificity compared to other phosphoinositides, for example, PLC‐δ‐PH domain, N‐terminal homology (ENTH) domain found in epsin proteins and the clathrin assembly synaptic protein AP180, and N‐terminal FERM domain found in ezrin/radixin/moesin proteins (ERM family). Second, proteins with unstructured domains containing significant basic residues that permit electrostatic interactions with PIP_2_, and therefore sequestration, but show less degree of PIP_2_ specificity, for example, MARCKS, growth‐associated protein 43 (GAP43), and cytoskeleton‐associated protein 23 (CAP23). These latter proteins are often termed as PIP_2_ buffers or PIPmodulins and are proposed to release PIP_2_ into the local environment following stimulation, allowing this source of PIP_2_ to act as an unmodified ligand.^9^ Most studies have investigated the role of MARCKS in regulating processes in VSMCs and VECs since it is a ubiquitously expressed protein, whereas GAP43 and CAP23 are mainly found in neurons.^1^

## 3 MARCKS

There is considerable knowledge about the chemical properties of MARCKS and fundamental cell signaling processes it is involved in, but relatively little is known about the functional outcome of MARCKS‐mediated signaling although it has been associated with neuronal development, cell migration, and proliferation, and secretary pathways, and peptide inhibitors against MARCKS are proposed to be an effective treatments for lung diseases and are currently in human clinical trials indicating it is druggable target.^14^, ^15^, ^16^

**FIGURE 1:**
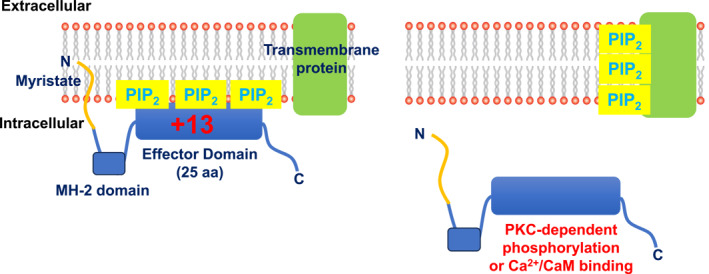
Molecular structure of MARCKS. Diagrammatic representation of MARCKS, showing that it is composed of three domains, an N‐terminal myristoylated domain which links to the plasma membrane, an MH‐2 domain, and a 25‐amino acid (aa) effector domain (ED) containing a +13 charge that produces electrostatic interactions with three PIP_2_ molecules in the intracellular leaflet of the plasma membrane. PKC‐dependent phosphorylation of serine residues or Ca^2+^‐CaM binding within the ED leads to the dispersion of the electrostatic interactions, with the release of PIP_2_ into the local environment that can bind to local transmembrane proteins and translocation of MARCKS from the plasma membrane into the cytoplasm.

MARCKS is a 32 KDa molecular weight protein which was first described as a protein kinase C (PKC) substrate, and since PKC is a hub for multiple signaling pathways, MARCKS was immediately recognized as a potentially significant protein.^14^, ^15^, ^17^, ^18^ MARCKS belongs to a family of unfolded proteins, MARCKS, MARCKSL1, MLP, and F52, with MARCKS characterized by three conserved domains: (1) N‐terminus domain containing a 24 amino acid sequence linked to myristic acid that enables anchoring of MARCKS into the plasma membrane, (2) MH‐2 domain of unknown function, and (3) central effector domain (ED) rich in basic lysine residues and also containing four serine residues that respectively provide positive charge of +13 for electrostatic interactions with PIP_2_ (likely 3 molecules) at the inner leaflet of the plasma membrane and PKC phosphorylation sites (Figure 1). It is important to note that interactions between the N‐terminal domain and the plasma membrane and lysine residues and PIP_2_ are both required to provide optimal MARCKS stability at the plasma membrane.^19^, ^20^ The ED acts as a PKC substrate and a Ca^2+^‐calmodulin (CaM)‐binding site, with both PKC‐dependent phosphorylation or Ca^2+^‐CaM binding at the ED reducing electrostatic interactions with PIP_2_, leading to PIP_2_ release into the local environment and MARCKS to be translocated into the cytosol due to its reduced stability at the plasma membrane.^13^, ^14^, ^15^, ^21^, ^22^ Interestingly, PKC‐dependent phosphorylation of ED reduces Ca^2+^‐CaM binding, whereas Ca^2+^‐CaM binding prevents PKC‐dependent phosphorylation.^14^, ^15^ These properties define MARCKS as a reversible PIP_2_ buffer, which can provide spatial sequestration and targeted release of PIP_2_. The release of PIP_2_ can produce bursts of PLC‐mediated IP_3_ and DAG or PI3K‐mediated PIP_3_ production, which indicates that MARCKS–PIP_2_ interactions protect PIP_2_ from PLC‐ and PI3K‐mediated hydrolysis.^23^, ^24^, ^25^

The MARCKS ED also acts as an actin‐binding site, which is reduced by PKC‐dependent phosphorylation or Ca^2+^‐CaM binding, suggesting that unstimulated MARCKS stabilizes the cytoskeleton, which can be reorganized by Ca^2+^‐CaM‐ and PKC‐dependent processes.^14^, ^15^

## 4 INVESTIGATING MARCKS

MARCKS knockout mice are embryonic lethal due to a dysfunctional central nervous system development,^26^, ^27^ although heterozygotic mice (MARCKS^+/−^) have been used to investigate reduced expression of MARCKS (e.g.,^28^). Therefore, molecular approaches used to study the role of MARCKS generally involve knockdown approaches with small interference RNA (siRNA) or morpholino oligonucleotide technologies (e.g.,^29^, ^30^). A successful pharmacological approach to study MARCKS has been the use of peptides raised against different domains of MARCKS. The selective MARCKS inhibitor MANS peptide is a 24 amino acid sequence that corresponds to the initial N‐terminal myristoylated region of MARCKS.^31^ MANS peptide competes with endogenous MARCKS for binding to the plasma membrane, leading to MARCKS being translocated into the cytosol and releasing PIP_2_ into the local environment. In addition, the hydrophobic myristate moiety means that MANS peptide is highly cell permeant, and a random sequence peptide can be used as a control.^31^ Overexpression of MARCKS ED peptides that can be either phosphorylated (based on endogenous MARCKS sequence), not phosphorylated (serine residues replaced with alanine), or more negatively charged (serine residues replaced with aspartate acid) are also useful strategies to assess MARCKS‐mediated processes (e.g.,^20^, ^32^).

When investigating MARCKS, it is important to remember that because MARCKS has an unfolded, nonglobular protein structure that reacts poorly with SDS molecules, and it often runs between 60 and 80 KDa on SDS‐page gels instead of its predicted 20–30 KDa molecular weight.^14^ Therefore, use of antigenic peptide control or knockdown approaches are important to consider when studying MARCKS expression using antibodies.

## 5 EXPRESSION AND INITIAL STUDIES OF MARCKS IN VSMCs

A PKC substrate protein with similar properties to MARCKS in rat brain was initially described using western blotting in rat and rabbit aortic lysates.^33^ Further western blotting studies have shown expression of MARCKS in vascular smooth muscle lysates or isolated VSMCs from rat and mouse aorta,^29^, ^34^ bovine carotid arteries,^35^ human coronary arteries,^36^, ^37^ ferret, rabbit, and mouse portal veins,^32^, ^38^ human saphenous vein,^37^ and rat and mouse mesenteric arteries.^30^ In addition, immunofluorescence studies have shown staining for MARCKS in the smooth muscle layer of human saphenous vein^37^ and mouse carotid arteries,^29^ and at, or close to, the plasma membrane of unstimulated VSMCs in ferret,^32^ rabbit and mouse portal vein,^38^ and rabbit and rat mesenteric arteries.^30^ These findings indicate that MARCKS is expressed in different vascular beds from different species and therefore is likely to have a central role in vascular function. Moreover, the stable anchoring of MARCKS at the plasma membrane in unstimulated VSMCs suggests that this is likely to be maintained through its interaction with PIP_2_.

In the first study to investigate a cellular role for MARCKS in VSMCs, Gallant et al^32^ examined the effect of MARCKS in regulating dynamic changes in CaM levels in ferret portal vein VSMCs following stimulation of PKC. Their findings showed that MARCKS and CaM co‐localized at the plasma membrane in unstimulated VSMCs but that following stimulation with a PKC agonist (the phorbol ester DPBA) MARCKS and CaM dissociated from each other and both co‐translocated to the cytosol. These DPBA‐mediated actions were prevented by over‐expression of a nonphosphorylatable MARCKS ED (ED4A) peptide acting as a decoy, in which the four serine residues present in the ED were replaced with alanine residues. Although this study did not investigate vascular function, it clearly indicated that MARCKS was a significant reservoir of CaM that can target the release of CaM through PKC‐dependent phosphorylation of its ED. Since both CaM and PKC are important signaling mediators in VSMCs, this study provided novel evidence for the importance of MARCKS in VSMCs. It should be noted that this study did not examine of these MARCKS‐mediated processes on the level or distribution of PIP_2_.

## 6 ROLE OF MARCKS IN PROLIFERATION AND MIGRATION OF VSMCs

There is significant evidence that MARCKS is involved in the development of intimal hyperplasia (IH), which is a major concerning factor in limiting arterial reconstruction through stenosis and thrombotic occlusion and is associated with VSMCs switching from a contractile to a synthetic phenotype with enhanced proliferation and migration. In microarray studies, MARCKS gene was shown to be upregulated in both prosthetic and vein grafts using a canine model,^39^, ^40^ which also fitted to previous findings showing that PKC is implicated in IH.^41^

### 6.1 Regulation of the cell cycle

In a series of works, the group of Monahan et al. showed that knockdown of MARCKS using siRNA arrested proliferation and reduced migration and motility of human coronary artery VSMCs.^29^, ^37^ Importantly, they demonstrated that knockdown of MARCKS using siRNA reduced the number of proliferating nuclei and neointimal formation in cultured segments of human saphenous vein^37^ and inhibited proliferating VSMCs and wall thickness in mouse aortic and femoral injury models.^29^, ^42^ Moreover, overexpression of MARCKS increased IH.^43^

**FIGURE 2:**
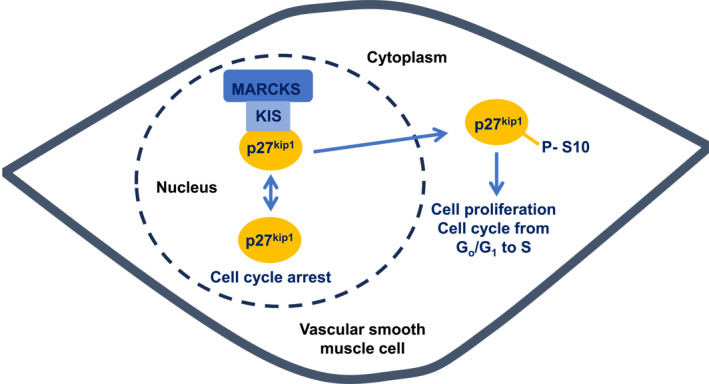
Proposed role of MARCKS in cell cycle and proliferation of VSMCs. MARCKS binds to and stabilizes KIS, which enables KIS to phosphorylate p27^kip1^ at serine 10. Phosphorylated p27^kip1^ is translocated from the nucleus to the cytoplasm, releasing a cell‐cycle brake leading to cell‐cycle progression and proliferation.

The cellular pathways underlying these MARCKS‐mediated changes in proliferation and migration of VSMCs are complex but seem to involve interactions between MARCKS, p27^kip1^, and kinase interacting with stathmin (KIS) (Figure 2). MARCKS knockdown is associated with an increase in p27^kip1^ levels, which is a cyclin‐dependent kinase inhibitor protein that acts as a critical cell‐cycle brake by preventing progression from G_o_/G_1_ to S phases. In support of findings, MARCKS knockdown‐mediated increases in proliferation and migration of VSMCs were reduced in p27^kip1−/−^ mice.^29^, ^42^ It is thought that MARCKS regulates p27^kip1^ by acting as an upstream modulator of KIS. MARCKS binds to KIS and prevents KIS being degraded through ubiquitination and proteasome processes, thus allowing KIS to phosphorylate p27^kip1^ at serine 10, which is then translocated from the nucleus to the cytoplasm. The removal of phosphorylated p27^kip1^ from the nucleus permits subsequent activation of cyclin‐dependent kinase proteins that drive cell‐cycle progression from G_o_/G_1_ to S phases. As such, MARCKS knockdown reduced phosphorylation of p27^kip1^, caused nuclear trapping of unphosphorylated p27^kip1^ and arrested the cell cycle, whilst reducing levels of KIS, cyclin D1 (Go/G1 phase‐related protein), and SKP2 (S phase‐related protein)^29^, ^42^ (Figure 2).

Interestingly, MARCKS knockdown had opposite actions on KIS in VECs, increasing KIS expression and cell proliferation.^29^, ^42^ These findings indicated that MARCKS has differential effects on KIS stability in VSMCs and VECs, with MARCKS increasing stability of KIS in VSMCs to increase proliferation and reducing stability of KIS in VECs to reduce proliferation. These results indicate that a MARCKS inhibitor may selectively reduce proliferation of VSMCs but not VECs. Clinically, this may aid prevention of IH formation whilst maintaining re‐endothelialisation, which is currently limited by treatments such as anti‐proliferation agents (e.g., sirolimus), mTOR inhibitors, and paclitaxel which have nonselective actions on VSMCs and VECs.^29^, ^42^ Maintaining re‐endothelialisation would be beneficial as this would likely reduce the risk of thrombus formation from an in‐stent restenosis.

A further study also showed that MARCKS is involved in inducing motility of VSMCs through providing a discrete pool of PIP_2_ at the plasma membrane, which is required for activation of the small GTPases Rac1 and Cdc42 and formation of lamellipodia and filopodia that are essential for motility.^43^

### 6.2 Regulation of TRPC1

Interestingly, the Ca^2+^‐permeable canonical transient receptor potential 1 (TRPC1) channel, which has been implicated in development of IH^44^ and requires PKC and PIP_2_ for activation in VSMCs,^45^, ^46^, ^47^, ^48^ is also regulated by MARCKS.^38^ MANS peptide activated TRPC1‐mediated whole‐cell and single channel currents in rabbit and mouse portal vein VSMCs, which were inhibited by lowering PIP_2_ levels with an anti‐PIP_2_ antibody and wortmannin (a PI4/5K inhibitor), and by reducing PKC‐dependent phosphorylation of TRPC1 proteins.^38^ Moreover, in resting VSMCs, MARCKS was shown to associate with TRPC1, and PIP_2_ was primarily bound to MARCKS and not TRPC1. Stimulation of VSMCs with MANS peptide, noradrenaline, and the phorbol ester PDBu induced dissociation of MARCKS and TRPC1, with PIP_2_ now binding more to TRPC1 than MARCKS.^38^ These findings proposed that stimulation of G‐protein‐coupled receptors leads to PKC‐dependent phosphorylation of TRPC1 proteins, which increases affinity for PIP_2_ that is released from MARCKS and acts as the channel activating ligand. It is also possible that PKC‐dependent phosphorylation of MARCKS contributes to this process by providing targeted PIP_2_ release for TRPC1 gating.

Taken together this evidence indicates that MARCKS is likely to be important in proliferation and migration of VSMCs and involved in processes linked to the development of IH. As such, MARCKS represents a legitimate therapeutic target to reduce arterial reconstruction associated with vascular grafts.

## 7 ROLE OF MARCKS IN VASCULAR CONTRACTILITY

In the background section in this review, we highlighted the importance of PIP_2_ as both a substrate for PLC‐ and PI3K‐mediated signaling pathways and as an unmodified ligand at ion channels and transporters in regulating vascular contractility. This paradox of PIP_2_ actions involving both hydrolysis and unmodified roles underlies the ideas of sequestration of PIP_2_ into independent pools at the plasma membrane. These ideas were further investigated by examining the role of MARCKS in mediating vascular contractility.^30^

We demonstrated that MANS peptide induced a concentration‐dependent contraction in rat and mouse mesenteric arteries, which had a similar magnitude to contractions evoked by the vasoconstrictors methoxamine (MO) and U46619 that act at α_1_‐adrenoceptors and thromboxane receptors, respectively.^30^ MANS peptide‐induced contractions were mediated by the activation of VGCCs, but were not associated with PLC‐mediated activity or membrane depolarization in contrast to MO which induced both PLC‐mediated activity and depolarization. MANS peptide also induced translocation of MARCKS from the plasma membrane to the cytosol but had little effect on the cellular distribution of the L‐type VGCC pore subunit protein CaV1.2, and reduced PIP_2_ binding to MARCKS whilst increasing PIP_2_ binding to CaV1.2. In addition, MANS peptide increased whole‐cell VGCC currents through shifting voltage‐dependence to more positive membrane potentials, which were prevented by lowering PIP_2_ with wortmannin. These results indicated that inhibition of MARCKS by MANS peptide and vasoconstrictor agents likely leads to the release of PIP_2_ from MARCKS into the local environment, where it binds to CaV1.2, inducing channel activation, increased Ca^2+^ influx, and contraction. This proposal has been previously suggested for the role of MARCKS in regulating activation of TRPC1 channel activity in VSMCs^38^ and thus these ideas may encompass a generalized picture of how MARCKS and PIP_2_ interact to regulate transmembrane proteins.

In comparison, our studies also showed that decreasing total MARCKS expression levels and cellular distribution of MARCKS at the plasma membrane using targeted morpholino oligonucleotides produced a pronounced reduction in contractility induced by MANS peptide, MO, and U46619.^30^ These opposing results suggest that acute inhibition of MARCKS, for example, with MANS peptide over several minutes, releases PIP_2_ to activate VGCCs and contraction, but that chronic knockdown of MARCKS, for example, following incubation with morpholinos for over 48 h, reduces MARCKS at the plasma membrane and therefore reduces an independent pool of PIP_2_ needed for targeted activation of VGCCs; hence, the reduction in contractility. The overall consequence of these ideas is that MARCKS inhibits contractility in unstimulated VSMCs and that vasoconstrictors cause disinhibition of these MARCKS‐mediated actions to induce contractility.

**FIGURE 3:**
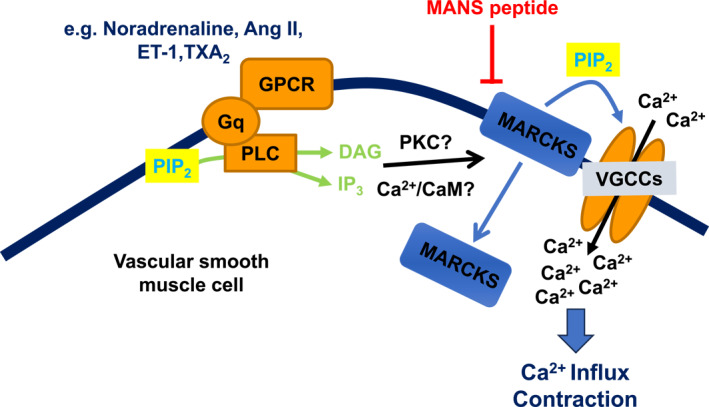
Proposed role of MARCKS in regulating contractility of VSMCs. Stimulation of G‐protein‐coupled receptors induces inhibition of MARCKS, likely via activation of PKC‐ and/or Ca^2+^‐CaM‐dependent pathways, which leads to the translocation of MARCKS from the plasma membrane to the cytoplasm and release of PIP_2_ that interacts with and stimulates VGCCs, leading to Ca^2+^ influx and contraction. This model suggests that in unstimulated VSMCs MARCKS inhibits the contractile process.

An important contrast in our findings is that although MANS peptide produced similar actions to vasoconstrictors upon MARCKS‐CaV1.2 interactions, MARCKS translocation, and changes in PIP_2_ binding to MARCKS and CaV1.2, they differed in their actions on membrane potential of VSMCs with vasoconstrictors producing a substantial membrane depolarization, whereas MANS peptide had a limited action.^30^ It is generally considered that vasoconstrictors induce contractility through inducing membrane potential depolarization through modulation of cation, Cl^−^, and K^+^ channels, which cause activation of VGCCs and Ca^2+^ influx.^6^ The present study poses important questions about these established processes, suggesting that in addition to membrane depolarization, vasoconstrictors may also cause disinhibition of MARCKS to directly activate VGGCs to produce contraction (Figure 3). As such, VGCCs become receptor‐operated channels at the resting membrane potential through the facilitatory effect of PIP_2_ released from MARCKS on VGCCs. This idea is not new; some 30 years ago, it was proposed that vasoconstrictors activate VGCCs held at resting membrane potentials.^49^

## 8 EXPRESSION AND FUNCTION OF MARCKS IN VECs

There is also substantial evidence that MARCKS is expressed in VECs, with immunoblots showing that MARCKS protein is expressed in cultured human umbilical vascular endothelial cells (HUVECs),^50^, ^51^ cultured rat cerebromicrovascular endothelial cells,^52^ cultured bovine pulmonary artery endothelial cells (BPAECs),^53^ cultured human coronary artery endothelium cells (HCAECs),^37^, ^42^ and cultured bovine aortic endothelial cells (BAECs).^54^, ^55^, ^56^, ^57^, ^58^ In addition, in unstimulated BAECs, MARCKS protein has been shown to be located at the plasma membrane using immunocytochemistry^55^ and expressed in the endothelium layer of murine carotid artery sections using immunohistochemistry.^57^

In BAECs, stimulation of PKC (with the phorbol ester PMA) induced a reduction in L‐arginine transport, an immediate substrate for NO synthesis, due to PKC inhibiting MARCKS and preventing interactions between MARCKS and the L‐arginine transporter CAT‐1.^54^ In addition, knockdown of MARCKS using antisense sequences reduced the action of PKC on L‐arginine transport. It was proposed that MARCKS may control distribution of CAT‐1 within selective areas of the plasma membrane of VECs, possibly areas linked to actin, to regulate effective L‐arginine transport, NO synthesis, and thus have an important role in controlling vascular tone.

**FIGURE 4:**
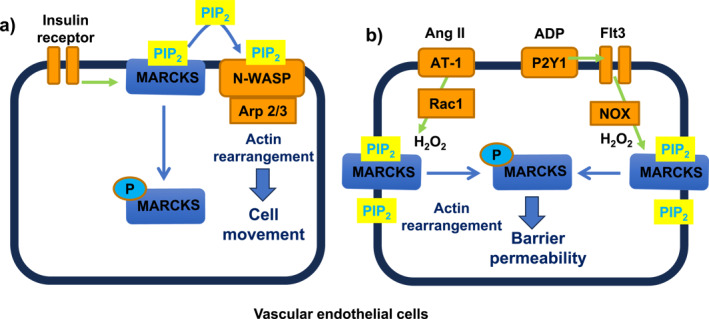
Proposed role of MARCKS in regulating movement of VECs and endothelial barrier permeability. (A) Stimulation of the insulin receptor leads to phosphorylation of MARCKS, which causes translocation of MARCKS from the plasma membrane into the cytosol and the release of PIP_2_ in the local environment where it binds to N‐WASP, leading to interactions with Arp 2/3 and increased actin arrangement and remodeling leading to cell movement. (B) Stimulation of AT‐1 and P2Y1 receptors leads to H2O2 production (via different Rac1 and NOX‐mediated pathways), which induces phosphorylation of MARCKS that is associated with increased actin arrangement, remodeling, and barrier permeability.

There is significant evidence from the group of Michel et al. that MARCKS has an important role in the movement of VECs and endothelial permeability.^55^, ^56^, ^57^, ^58^ Knockdown of MARCKS with siRNA inhibited movement of BAECs using a wound healing assay,^55^ and further studies indicated that phosphorylation of MARCKS involves multiple signaling pathways, which are likely to converge on actin‐mediated processes to alter focal adhesion areas and cell movement.^55^, ^56^, ^57^, ^58^ Insulin‐induced MARCKS phosphorylation increased local PIP_2_, which bound to the PIP_2_‐binding protein N‐WASP to mediate Arp 2/3 and actin activation and increased cell movement^55^ (Figure 4A). Moreover, the reactive oxygen species H_2_O_2_ has been shown to have a central role in MARCKS‐mediated phenotypes in VECs, through increasing MARCKS phosphorylation and endothelial permeability.^56^ Moreover, production of H_2_O_2_ and actin reorganization through stimulation of AT_1_ receptors by angiotensin II (Ang II)^57^ and P2Y1 receptors by adenosine diphosphate (ADP)^58^ was associated with induced MARCKS phosphorylation through activation of the cytoskeletal‐associated Rho GTPase Rac1 and a tyrosine kinase receptor (Flt3)‐NADPH oxidase (NOX)‐linked pathway, respectively (Figure 4B).

## 9 FUTURE WORK

This review discusses a growing body of evidence showing that MARCKS is involved in multiple signaling pathways in VSMCs and VECs, which impact diverse functions such cell proliferation and migration, endothelial barrier permeability, and smooth muscle contractility. As such, even though our current understanding on the importance of MARCKS in the vasculature is only at its infancy, it is likely that MARCKS and its associated signaling pathways are legitimate future therapeutic targets for vascular diseases such as atherosclerosis, systemic and pulmonary hypertension, and sepsis.

What might be useful next steps? To date, the role of MARCKS have been mostly carried out using in vitro studies, cultured VSMCs and VECs, and freshly isolated VSMCs and vessel segments from different animal species. It will be important to expand these studies into investigating the role of MARCKS using animal models of disease such as examining changes in MARCKS‐mediated signaling and function in vascular preparations from systemic and pulmonary artery hypertensive models, for example, spontaneous hypertensive rats, Ang II‐induced hypertensive mice, hypoxia‐ or monocrotaline‐induced pulmonary hypertension rats. A useful template might be the recent interesting findings proposing that regulation of N‐myristylation, MARCKS, and PIP_2_ levels are associated with cardiac hypertrophy and failure.^59^, ^60^ Of course, further studies using human tissue will also be important to validate results from these animal models.

The proposal that MARCKS may mediate receptor‐operated activation of CaV1.2 VGCC channels in VSMCs to induce Ca^2+^ influx and contractility of VSMCs, without a requirement for membrane depolarization, is a potential paradigm shift in our understanding of vascular contractility and control of vascular tone and blood pressure. It is possible that excessive increases or decreases in MARCKS‐mediated vascular contractility may contribute to vascular diseases associated with profound changes in vascular tone such as hypertension and sepsis. These ideas are essential topics for investigation if we are gain a significant understanding of the role and importance of MARCKS in vascular contractility.

Since pharmacological regulation of MARCKS is achievable through selective peptides,^14^, ^16^ which are currently in human clinical trials for lung disease, it is likely that modulation of MARCKS using these peptides in *in vivo* studies may be feasible and that these studies might not only offer important insights into potential cardiovascular side effects of these peptides when treating lung diseases and but also the potential effectiveness of these peptides in regulating cardiovascular parameters and vascular disease.

## 10 SUMMARY

MARCKS regulates multiple signaling molecules and processes in VSMCs and VECs, which have profound effects on vascular physiological and pathological functioning, including those involving PIP_2_‐, PKC‐, Ca^2+^‐CaM‐, and H_2_O_2_‐mediated pathways. As such, MARCKS and its associated signaling pathways are likely to represent future therapeutic targets for vascular disease.

## AUTHOR CONTRIBUTIONS

Anthony P. Albert wrote the manuscript. All authors critically advised and agreed to the final submitted article.

## CONFLICT OF INTEREST STATEMENT

The authors declare that they have no competing interests.

## ETHICS STATEMENT

All animal procedures were carried out in accordance with guidelines laid down by City St. George's, University of London Animal Welfare Committee, and conform with the principles and regulations described by the Service Project License: 70/8512.

## Data Availability

The data that support the findings of this study are available from the corresponding author upon reasonable request.
